# White matter microstructure mediates the association between physical fitness and cognition in healthy, young adults

**DOI:** 10.1038/s41598-019-49301-y

**Published:** 2019-09-09

**Authors:** Nils Opel, Stella Martin, Susanne Meinert, Ronny Redlich, Verena Enneking, Maike Richter, Janik Goltermann, Andreas Johnen, Udo Dannlowski, Jonathan Repple

**Affiliations:** 1 Department of Psychiatry, University of Münster, Münster, Germany; 2 Department of Economics, University of Münster, Münster, Germany; 3 Department of Neurology, University of Münster, Münster, Germany

**Keywords:** Risk factors, Cognitive neuroscience

## Abstract

We aimed to extend our knowledge on the relationship between physical fitness (PF) and both white matter microstructure and cognition through in-depth investigation of various cognitive domains while accounting for potentially relevant nuisance covariates in a well-powered sample. To this end, associations between walking endurance, diffusion-tensor-imaging (DTI) based measures of fractional anisotropy (FA) within brain white matter and cognitive measures included in the NIH Toolbox Cognition Battery were investigated in a sample of n = 1206 healthy, young adults (mean age = 28.8; 45.5% male) as part of the human connectome project. Higher levels of endurance were associated with widespread higher FA (p_FWE_ < 0.05) as well as with enhanced global cognitive function (p < 0.001). Significant positive relationships between endurance and cognitive performance were similarly found for almost all cognitive domains. Higher FA was significantly associated with enhanced global cognitive function (p < 0.001) and FA was shown to significantly mediate the association between walking endurance and cognitive performance. Inclusion of potentially relevant nuisance covariates including gender, age, education, BMI, HBA1c, and arterial blood pressure did not change the overall pattern of results. These findings support the notion of a beneficial and potentially protective effect of PF on brain structure and cognition.

## Introduction

Over recent years increased attention has been dedicated to the relationship between physical fitness (PF) and brain physiology. PF has repeatedly shown protective effects on diverse mental health outcomes including decreased risk of future dementia^1^, decreased stress-related^2^ and depressive symptoms^2,3^. A better understanding of the precise relationships between PF as a modifiable lifestyle factor and healthy brain physiology would provide insights of crucial relevance for the future development of preventive efforts for various neuropsychiatric disorders.

More specifically, meta-analytic evidence supports the notion of a positive relationship between measures of physical activity and brain functioning as measured by cognitive performance across a wide range of cognitive domains including executive function, attention and working memory^4,5^. Importantly, these associations have been demonstrated across the life-span^5,6^ in healthy samples as well as in samples showing differing degrees of cognitive impairment (mild cognitive impairment, dementia)^6–8^.

Corresponding findings from neuroimaging research on positive associations between PF and white matter (WM) structure^9,10^ as well as gray matter volume^11,12^ appear to further support the notion of neuroprotective effects of physical activity and fitness. However, considerably less is known on the positive association of PF with white matter microstructure^13,14^ as assessed by diffusion tensor imaging (DTI), and studies combining PF, DTI and cognition are rare: In older adults (>60 years), white matter microstructure was positively associated with cardiorespiratory fitness and working memory performance^15^. The aforementioned correlational studies are furthermore supplemented by findings of prospective associations between PF, brain structure and cognition from the few existing longitudinal studies: Findings from one of the few randomized controlled trials (RCT) involving 120 participants on this topic reported increased hippocampal gray matter volume and increased cognitive performance compared to the control group following a 12-month aerobic walking intervention^16^. Similarly, another RCT adds further weight to this notion by showing that improvements in PF after a 12-month exercise intervention were associated with increased temporal and prefrontal white matter integrity as assessed via fractional anisotropy (FA) as well as with increased memory performance in an elderly sample (age 55–80; n = 70)^17^.

In sum, previous neurobiological research has demonstrated a positive relationship between PF and both brain structure and cognitive function. Yet, some heterogeneity in the literature must be acknowledged: As pointed out by a recent review and meta-analysis the association between aerobic exercise and cognitive performance varied substantially with effect sizes ranging from g = −0.015 to 0.275 for executive function and from g = 0.052 to 0.146 for attention and processing speed depending on the selected studies and the type of assessment instrument^18^. It thus appears highly relevant to further clarify the contribution of PF on distinct cognitive domains in a well powered sample. Furthermore, the observed heterogeneity might partially be due to the potentially undiscovered contribution of relevant covariates. Since the majority of previous studies did not control for further socioeconomic, cardiovascular and metabolic risk factors in a standardized manner, the possibility that reported associations between PF and brain structure and cognition in previous research might be subject to bias or spurious correlations cannot be suspended up to now. This notion appears particularly relevant since several variables closely related to PF such as body mass index (BMI)^19,20^, blood pressure^21^ as well as metabolic serum markers^22^ but also presence and history of neuropsychiatric disorders^23–25^ have been shown to contribute to variation in both brain structural integrity and cognitive function. Moreover, the majority of previous studies have investigated single cognitive domains only, while studies investigating associations between PF, white matter integrity and multiple differential cognitive domains simultaneously are rare which makes it difficult to delineate and to compare domain specific contributions of PF. Therefore, the aim of the present work was to overcome limitations of previous research (a) by validation of previous findings on the association between PF, brain structural integrity and cognition in a large sample of healthy, young adults (b) by simultaneously investigating the contribution of PF to a wider range of cognitive domains, (c) by accounting for potentially relevant covariates and (d) by performing mediation analyses to investigate whether the PF-cognition association is mediated by white matter microstructure.

## Material and Methods

### Participants

We investigated open-access brain imaging data from the Human Connectome Project (HCP) WU-Minn HCP 1200 Subjects Data Release^26^ (for further information on details of data acquisition and processing in this sample please see: https://www.humanconnectome.org/study/hcp-young-adult/document/1200-subjects-data-release). Exclusion criteria were neurodevelopmental disorders (e.g., autism), documented neuropsychiatric disorders (e.g., schizophrenia or depression), neurologic disorders (e.g., Parkinson’s disease), diabetes or high blood pressure^26^. Per HCP protocol, all subjects gave written informed consent to the Human Connectome Project consortium. All subject recruitment procedures and informed consent forms, including consent to share data, were approved by the Washington University Institutional Review Board^27^. All experiments were performed in accordance with relevant guidelines and regulations. All 1206 subjects were included in the present study. Analyses were performed with the maximum number of available data for each analysis. We report the respective n for each analysis. The mean age of the sample was 28.8 years, 45.5% of all participants were male. BMI was calculated as the body weight (in kilogram) divided by squared body height (in meters) (mass_kg_/height_m_^2^). BMI was available for n = 1200, the mean was 27.09. Total education years, a proxy for socioeconomic status, were calculated as the years of completed education (range 11–17 years, mean = 14.87 years). Subjects were primarily recruited in Missouri; additional recruiting efforts were made to ensure that participants generally reflect the ethnic and racial composition of the U.S. population. For data acquisition participants visited the Washington University, St. Louis, Missouri, twice with a fixed order of magnetic resonance imaging (MRI) scanning and extensive behavioral assessment on each day^26^.

### Endurance

Physical fitness was operationalized as a result on a walking endurance test. Endurance data was available for n = 1204 subjects. Endurance was assessed using the 2-min walk test as part of the Motor domain of the official NIH toolbox for the Assessment of Neurological and Behavioral Function^28^. In a single trial, subjects were asked to walk as fast as they could for 2 minutes on a 50-foot (out and back) course. After two minutes distance was measured in feet and inches. The raw scores were normed to a scale score with mean = 100, SD = 15. Extensive reliability and validity tests were performed for this test during implementation of the NIH toolbox^29^, showing a good test-retest reliability (intraclass correlation (ICC) > 0.80) and very high external validity, for instance a high correlation with the 6 minute walk test (r > 0.96). Performance on these walking tests have been associated with functional, morbidity and mortality outcomes across all ages both in clinical as well as healthy populations^30^.

### Cognitive performance

Cognitive measures were available for n = 1187 subjects. All tests and cognitive outcome parameters included in the HCP dataset were included in the study. The NIH Cognition Total Composite Score (“global cognition score”) is calculated as the average of subtest-scores contained in the NIH Toolbox Cognition Battery (Flanker, Dimensional Change Card Sort, Picture Sequence Memory, List Sorting and Pattern Comparison, Picture Vocabulary and Reading Recognitions) and reflects overall cognitive performance^28,31,32^. For further information on all cognitive measures see Supplementary Methods 1.

### DTI data acquisition

The following methods (Section 2.4 and 2.5) have been described in detail in our previous work^19^. In brief, DTI data was available for n = 1050 in the HCP sample. Data for the HCP was acquired on a customized Siemens 3 T “Connectome Skyra” housed at Washington University in St. Louis, using a standard 32-channel Siemens receive head coil and a “body” transmission coil designed by Siemens specifically for the smaller space available using the special gradients of the WU-Minn and MGH-UCLA Connectome scanners^33,34^.

A full diffusion MRI session includes 6 runs (each approximately 9 minutes and 50 seconds), representing 3 different gradient tables, with each table acquired once with right-to-left and left-to-right phase encoding polarities, respectively. Each gradient table includes approximately 90 diffusion weighting directions plus 6 b = 0 acquisitions interspersed throughout each run. Diffusion weighting consisted of 3 shells of b = 1000, 2000, and 3000 s/mm^2^ interspersed with an approximately equal number of acquisitions on each shell within each run (Sequence: Spin-echo EPI, TR 5520 ms, TE 89,5 ms, flip angle 78 deg, refocusing flip angle 160 deg, FOV 210 × 180 (RO × PE), matrix 168 × 144 (RO × PE), slice thickness 1.255 mm, 111 slices, 1.25 mm isotropic voxels, multiband factor 3, echo spacing 0.78 ms, BW 1488 Hz/Px, phase partial fourier 6/8, b-values 1000, 2000, 3000 s/mm^2^)^34,35^.

### DTI data preprocessing and analysis

Diffusion data accessible from the HCP was preprocessed with their MR Diffusion Pipeline^36^: The diffusion preprocessing pipeline does the following: normalizes the b0 image intensity across runs; removes EPI distortions, eddy-current-induced distortions, and subject motion; corrects for gradient-nonlinearities; registers the diffusion data with the structural; brings it into 1.25 mm structural space; and masks the data with the final brain mask: 1. Basic preprocessing: Intensity normalization across runs, preparation for later modules. 2. ‘TOPUP’ algorithm for EPI distortion correction. 3. ‘EDDY’ algorithm for eddy current and motion correction. 4. Gradient nonlinearity correction, calculation of gradient bvalue/bvector deviation. 5. Registration of mean b0 to native volume T1w with FLIRT BBR + bbregister and transformation of diffusion data, gradient deviation, and gradient directions to 1.25 mm structural space. The brain mask is based on FreeSurfer segmentation.

Tract-based spatial statistics (TBSS)^37^ is a well-established analysis method for DTI imaging and was applied as described extensively in a recent study^19^. Briefly, standard TBSS preprocessing was performed^37^: The FA images were registered to the FMRIB58 FA template and averaged to create a mean FA image. A WM skeleton was created with an FA threshold of 0.2 and overlaid onto each subject’s registered FA image. Individual FA values were warped onto this mean skeleton mask by searching perpendicular from the skeleton for maximum FA values.

To test for statistical significance, we used the non-parametric permutation testing implemented in FSL’s ‘randomize’ with 5000 permutations. Threshold-Free Cluster Enhancement (TFCE)^38^ was used to correct for multiple comparisons. This allows to estimate cluster sizes corrected for the family-wise error (FWE; p < 0.05, 5000 permutations). MNI coordinates and cluster size at peak voxel were derived with FSL Cluster and the corresponding WM tract retrieved from the ICBM-DTI-81 white-matter atlas^39^.

### Statistical analyses

1. In order to investigate whether associations between endurance and cognitive measures exist, we performed hierarchical linear regressions using SPSS (IBM Version 25). As baseline, the global cognition score was regressed on endurance. Control variables (sex, age, years of completed formal education, BMI, HbA1c and systolic blood pressure) were then successively incorporated to extend the regressions in order to check if any of the control variables significantly weaken the estimated observations. Subjects with missing values for any of the control variables were dropped beforehand to ensure a continuous sample throughout all hierarchical model specifications. The resulting sample consisted of n = 801 observations. The results are reported in Supplementary Results 1.
  In the next step, we investigated whether endurance has a specific impact on any of the domains of cognition. Thus, we linearly regressed scores of each cognitive domain on endurance as the sole independent variable (“baseline”) as well as on the full model containing all above-mentioned covariates. The results are reported in Table 1.

**Table 1:** Regression analyses of endurance and FA with cognitive subscores.

		Baseline regression Endurance	Full model regression Endurance^a^	Baseline regression FA	Full model regression FA^a^
Picture Sequence Memory: [non-verbal] episodic memory	ß	0.510**	0.109**	0.158**	0.119**
	p-value	0.000	0.002	0.000	0.001
	df	1187	800	1048	714
Dimensional Change Card Sort Test: executive function & cognitive flexibility	ß	0.574**	0.155**	0.054	0.038
	p-value	0.000	0.000	0.080	0.316
	df	1187	800	1048	714
Flanker Inhibitory Control and Attention Test: executive function	ß	0.524**	0.132**	0.021	0.020
	p-value	0.000	0.000	0.503	0.593
	df	1187	800	1048	714
Penn Progressive Matrices, total correct responses: fluid intelligence	ß	0.735**	0.171**	0.071*	0.073
	p-value	0.000	0.000	0.021	0.051
	df	1187	800	1048	714
Oral Reading Recognition Test: reading decoding skills	ß	0.518**	0.119**	0.084**	0.065
	p-value	0.000	0.001	0.007	0.083
	df	1178	800	1044	714
Picture Vocabulary Test: vocabulary knowledge	ß	0.711**	0.116**	0.087**	0.071
	p-value	0.000	0.001	0.005	0.059
	df	1187	800	1048	714
Pattern Comparison Processing Speed Test	ß	0.574**	0.145**	0.072*	0.057
	p-value	0.000	0.000	0.020	0.126
	df	1187	800	1048	714
Delay Discounting, 40k: Self-regulation/Impulsivity	ß	0.210**	0.083*	0.082**	0.035
	p-value	0.000	0.019	0.008	0.353
	df	1179	800	1045	714
Variable Short Penn Line Orientation, total correct items: spatial orientation processing	ß	0.448**	0.129**	0.016	−0.002
	p-value	0.000	0.000	0.608	0.947
	df	1178	800	1045	714
Short Penn Continuous Performance Test, specificity of right decisions: Sustained attention	ß	0.222**	0.085*	0.096**	0.023
	p-value	0.000	0.016	0.002	0.541
	df	1179	800	1045	714
Penn Word Memory Test: verbal episodic memory	ß	0.312**	0.083*	0.131**	0.117**
	p-value	0.000	0.018	0.000	0.002
	df	1179	800	1045	714
List Sorting Working Memory Test	ß	0.582**	0.064	0.059	0.098**
	p-value	0.000	0.070	0.056	0.009
	df	1187	800	1048	714

^a^Full regression model controls for age, sex, education years, BMI HbA1c, Systolic Blood Pressure.

Endurance: This test measures sub-maximal cardiovascular endurance by recording the distance that the participant is able to walk on a 50-foot (out and back) course in 2 minutes. The participant’s raw score is the distance in feet and inches walked in 2 minutes. The raw scores are normed to a scale score with mean = 100, 1 SD = 15.

FA: Fractional anisotropy, mean value extracted from the FA-global cognition association results mask.

ß: Standardized ß-Coefficients from regression analyses with the cognitive scores as the dependent variables.

df: degrees of freedom, all available data was used for the respective analyses leading to different degrees of freedom for different models.

For more information on the cognitive subscores see Supplementary Methods 1.
2. Second, we investigated whether endurance has any impact on FA across the entire WM skeleton using general linear models within FSL. We accounted for the effects of nuisance covariates that could influence WM structure: age^40^ and sex^41^. Combined endurance and DTI data were available for a sample of n = 1048 subjects. To further test for the specificity of endurance-related effects, we extracted a mean FA value from all significant voxels and used this value in a baseline regression of endurance on FA within SPSS. In analogy to 1., we employed hierarchical regression specifications, subsequently adding control variables (age, sex, education years, BMI, HbA1c and systolic blood pressure) to the model. The results are reported in Supplementary Results 1.
3. We sought to investigate the relationship between cognition and endurance-related white matter microstructure. In line with the analysis in steps 1 and 2 above, hierarchical regressions within SPSS were performed using global cognition as the dependent variable and the extracted FA value as the regressor of interest, subsequently adding the above-mentioned covariates in the same order. The results are reported in Supplementary Results 1. Furthermore, additional regression analyses between FA and all available measures of cognitive domains were carried out to further delineate the contribution of specific domains to the observations made in step 1. above. Thus, we linearly regressed these subscores on FA as the sole independent variable (“baseline”) as well as on the full model containing all above-mentioned covariates. The results are reported in Table 1.
4. To directly test the presence of an indirect effect of walking endurance on cognitive performance through white matter microstructure, we carried out a mediation analysis with walking endurance as predictor variable (X), white matter microstructure as mediator (M) and global cognitive performance as outcome variable (Y). For this analysis step, a bootstrapping approach as implemented in the SPSS macro PROCESS was applied (http://www.processmacro.org) which has been demonstrated to provide reliable results in neuroimaging research^42–44^. PROCESS estimates direct and indirect effects between a defined set of variables by applying an ordinary least squares path analytic framework. Inference of indirect (mediated) effects is assessed through bootstrap confidence intervals. Significance of an indirect effects is assumed if the 95% confidence interval (95%-CI) does not include zero. The number of bootstrap samples was set to n = 5000. Unstandardized regression coefficients (coeff) and standard errors (SE) are presented for each effect. To enhance comparability with the literature, mediation analyses were repeated using standardized (z-transformed) variables in order to obtain standardized regression coefficients (Std coeff). In line with all other analyses steps the mediation analysis was first conducted without any covariates and secondly repeated by including age, sex, BMI, education, HbA1c and systolic blood pressure as nuisance regressors in the model.

## Results

### Endurance – cognition

Endurance was positively associated with the global cognition score (degrees of freedom (df) = 800, standardized coefficient ß = 0.338, p < 0.001, explained variance of the model R^2^ = 0.115; Supplementary Fig. 1). After adding age, sex, education years, BMI, HbA1c and systolic blood pressure as regressors, endurance was still positively associated with cognition (df = 793, ß = 0.229, p < 0.001, R^2^ = 0.225). While the (subsequent) inclusion of additional regressors improved the share of variance explained by the model, only the inclusion of years of education had a significant impact on the estimated effect of endurance on cognition. For all details on the hierarchical regression analyses, please see Supplementary Results 1. The regressions of all cognition subscores on endurance revealed significant associations of the latter with all cognitive measures except for the list sorting working memory task which marginally failed to reach significance in the full model controlling for all covariates (ß = 0.064, p = 0.070). Most pronounced associations between endurance and cognitive performance emerged for cognitive flexibility (ß = 0.155, p < 0.001), fluid intelligence (ß = 0.171, p < 0.001) and cognitive processing speed (ß = 0.155, p < 0.001) when correcting for age, sex, education years, BMI, HbA1c and systolic blood pressure. For more details on the association with all subtests see Table 1.

### Endurance – white matter

**Figure 1:**
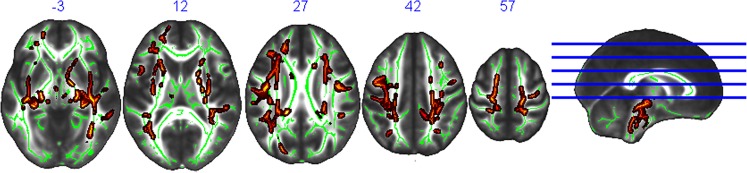
Positive Association of Endurance with Fractional Anisotropy. Top: Axial slices with corresponding y-axis values (MNI) are presented. Red-yellow areas represent voxels (using FSL’s “fill” command for better visualization), where a significant positive association between Endurance and Fractional Anisotropy was detected (pFWE < 0.05, corrected for age and sex). Sagittal view with blue lines indicating axial slices on the left.

We found a significant positive association (p_FWE_ < 0.05; cluster size k: 27313 in 4 clusters) between FA and endurance in large, widespread clusters, including the genu of the corpus callosum, the bilateral longitudinal superior fascicle, the bilateral internal and external capsule, the bilateral uncinate fascicle, the corticospinal tract and the cerebellar peduncles among others (Table 2, Fig. 1, Supplementary Fig. 2). Hierarchical regression analyses revealed a positive association between endurance and extracted FA values even after correcting for all additional regressors (df = 723, ß = 0.145, p < 0.001, R^2^ = 0.155). For all details on the hierarchical regression analyses, please see Supplementary Results 2.

**Table 2:** Positive association of Fractional Anisotropy with Endurance.

Voxels	*p*_*FWE*_	MNI X (mm)	MNI Y (mm)	MNI Z (mm)
26380	0.002	8	−40	−36
685	0.037	−19	44	11
185	0.040	−20	−81	24
63	0.049	−15	42	29
**Probabilities of affected tracts in percent**				
**Region**			**Laterality**	**Probability**
Middle cerebellar peduncle:				4.25
Pontine crossing tract (a part of MCP)				1.04
Body of corpus callosum				1.60
Splenium of corpus callosum				0.30
Corticospinal tract			R	1.16
Corticospinal tract			L	1.18
Medial lemniscus			R	0.65
Medial lemniscus			L	0.68
Inferior cerebellar peduncle			R	0.30
Inferior cerebellar peduncle			L	0.46
Superior cerebellar peduncle			R	0.91
Superior cerebellar peduncle			L	0.77
Cerebral peduncle			R	1.45
Cerebral peduncle			L	1.88
Anterior limb of internal capsule			R	0.95
Anterior limb of internal capsule			L	0.33
Posterior limb of internal capsule			R	0.85
Posterior limb of internal capsule			L	0.36
Retrolenticular part of internal capsule			R	0.87
Retrolenticular part of internal capsule			L	0.41
Anterior corona radiata			R	0.59
Anterior corona radiata			L	0.62
Superior corona radiata			R	1.85
Superior corona radiata			L	1.76
Posterior corona radiata			R	1.16
Posterior corona radiata			L	1.25
Posterior thalamic radiation (include optic radiation)			R	1.08
Posterior thalamic radiation (include optic radiation)			L	0.62
Sagittal stratum (include inferior longitudinal fasciculus and inferior fronto-occipital fasciculus)			R	1.32
Sagittal stratum (include inferior longitudinal fasciculus and inferior fronto-occipital fasciculus)			L	0.42
External capsule			R	2.34
External capsule			L	2.31
Fornix (cres)/Stria terminalis			R	0.76
Fornix (cres)/Stria terminalis			L	0.86
Superior longitudinal fasciculus			R	1.52
Superior longitudinal fasciculus			L	2.48
Superior fronto-occipital fasciculus (could be a part of anterior internal capsule)			R	0.13
Superior fronto-occipital fasciculus (could be a part of anterior internal capsule)			L	0.01
Uncinate fasciculus			L	0.10
Tapetum			R	0.01
Unclassified				58.18

Negative correlation.

No significant results.

On the top dimensions of clusters (number of voxels) and localization of signal peaks (MNI coordinates) are given for regions showing maximal differences of tract-based spatial statistics values (signal peak). Below are the white matter tracts in the cluster based on the JHU ICBM-DTI-81 White-Matter Labels (as implemented in FSL).

Probabilities of affected tracts: It gives the (average) probability of all significant voxels being a member of the different labelled regions within the atlas (JHU ICBM-DTI-81 White-Matter), calculated with the FSL tool “atlasquery”.

### White matter – cognition

FA and global cognition were positively associated (df = 723, ß = 0.132, p < 0.001, R^2^ = 0.017), even after correcting for all additional regressors (df = 716, ß = 0.116, p < 0.001, R^2^ = 0.183). For all details on the hierarchical regression analyses, please see Supplementary Results 3. The regression analyses of all subscores revealed the strongest association for non-verbal episodic memory (ß = 0.119, p = 0.001) and verbal episodic memory (ß = 0.117, p = 0.002) and a trend in fluid intelligence (ß = 0.021, p = 0.051) when correcting for age, sex, education years, BMI, HbA1c and systolic blood pressure. For more details on the association with different subtests see Table 1.

### Mediation analysis

**Figure 2:**
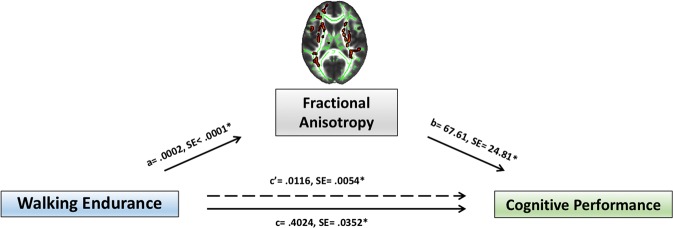
Fractional anisotropy mediates the association between walking endurance and cognitive performance. Depiction of the applied mediation model: Unstandardized coefficients and standard errors for each path of the mediation model are presented. Note that c represents the direct effect and c‘ the indirect effect. * indicates significance at p < 0.05.

In line with analyses steps 3.1–3.3 the mediation model confirmed a significant association between walking endurance and FA (Std coeff = 0.1217, coeff = 0.0002, SE < 0.0001, 95%-CI = 0.0001 to 0.0003, t = 3.93, p = 0.0001) as well as a significant association between FA and global cognitive performance (Std coeff = 0.0780; coeff = 67.61, SE = 24.81, 95%-CI = 18.93 to 116.29, t = 2.73, p = 0.0065). The mediation model furthermore yielded a significant positive indirect (mediated) effect of walking endurance on cognitive performance through FA (indirect effect: Std coeff = 0.0095; coeff = 0.0116, SE = 0.0054, 95%-CI = 0.0025 to 0.0236) (see Fig. 2). Moreover, a significant direct effect of walking endurance on cognitive performance could also be detected (direct effect: Std coeff = 0.3302; coeff = 0.4024, SE = 0.0352, 95%-CI = 0.3334 to 0.4714, t = 11.44, p < 0.001). The mediation model including age, sex, BMI, HbA1c, education years and blood pressure as additional nuisance covariates confirmed these results and similarly yielded a significant indirect (mediated) effect of walking endurance on cognitive performance through FA (indirect effect: Std coeff = 0.0129; coeff = 0.0157, SE = 0.0079, 95%-CI = 0.0026 to 0.0339).

## Discussion

With the present work we provide evidence for a positive relationship between PF and both white matter microstructure as well as cognitive performance in a large sample of healthy young adults. The observed positive association between PF and cognitive function extended to nearly all cognitive domains with most pronounced associations for fluid intelligence, cognitive flexibility and processing speed. Our finding of a significant mediation effect of PF on cognitive performance through white matter microstructure furthermore points to a crucial role of brain structural alterations in the association between PF and cognition. Importantly, the observed significant associations between PF, brain structure and cognition withstood correction for a wide range of potentially relevant nuisance covariates, which makes it unlikely that the observed pattern of results was biased by the presence of common cardiovascular or metabolic risk factors. A setup of hierarchical regressions revealed that while regressors such as age, sex, BMI and HbA1c were associated with cognition and FA in the respective models, only the inclusion of education years substantially weakened the estimated effect of endurance on cognition and FA. The present study thus supports the concept of a robust positive relationship between PF and preserved brain structural integrity as well as cognitive performance in a wide range of cognitive domains.

The presented positive association between PF as operationalized through walking endurance and overall cognitive function is in line with reports on a similar positive relationship between physical activity or fitness and cognition from the literature including meta-analyses^4,8,16^. Two aspects related to the association between PF and cognitive performance in the present study warrant further discussion: First, the present findings were based on analyses in a relatively young sample of healthy adults and thus demonstrate that associations between PF and cognitive performance are already present during early adulthood. This finding is supported by previous research reporting a corresponding positive relationship between PF and cognition across the life-span that appears to be similarly present in children and adolescents as well as in older healthy adults^5,6^.

Second, the present study included data on a broad spectrum of cognitive domains that were simultaneously investigated and revealed positive associations between endurance and cognitive performance in a wide range of cognitive domains with strongest effect sizes for associations with cognitive flexibility, processing speed and fluid intelligence. This finding appears to be in line with previous meta-analytic evidence on similar associations between physical activity and executive function and cognitive flexibility^5^. Of note, while the significant associations between endurance and cognition were demonstrated for nearly all cognitive domains and could be demonstrated even when controlling for the presence of further metabolic risk factors, the association between endurance and performance in the list sorting working memory task marginally failed to reach significance when controlling for further covariates. Importantly, this finding might suggest that in comparison to other cognitive domains working memory performance in young adults is more likely to be influenced by other metabolic risk factors such as HbA1c, BMI or blood pressure, a finding that is in line with a previously reported pronounced association between BMI and working memory performance^45^.

Another important finding of the present work was the positive relationship between endurance and fractional anisotropy as a measure of white matter integrity. While evidence on grey and white matter volume associations with PF are well-established^11^, investigations into PF-FA relationship is scarce. However, these results correspond to previous studies demonstrating positive correlations between physical exercise and white matter microstructure^15,17^. This association may be mediated by several neurobiological pathways (for reviews see^9,46^): higher PF might lead to better WM microstructure via increased cerebrovascular health and perfusion^47,48^, neuroplasticity-related effects (e.g. BDNF excretion)^49^, oligodendrocyte proliferation^50^ and neuroprotective effects via down-regulation of the inflammatory system^51,52^.

Importantly, additional investigation of cognitive domains furthermore indicated that the observed significant relationship between cognitive performance and PF-associated white matter microstructure was primarily driven by associations between episodic memory and white matter microstructure supporting previous studies into white matter integrity associations with this specific cognitive domain^53,54^. Also, this suggests white matter integrity as one potential means by which physical exercise may augment episodic memory. In contrast, other cognitive measures such as performance in attention related tasks did not show significant associations with white matter microstructure in PF-related white matter tracts in the present study. This finding is of relevance for future studies that seek to investigate neural correlates of cognition and might furthermore explain the heterogeneous findings for the association between cognition and white matter microstructure from the literature^55^.

Taken together, while we fully acknowledge the cross-sectional character of the present study, the observed pattern of results appears to support the notion of a beneficial effect of PF on cognitive function, possibly mediated by its effect on white matter integrity. This is supported by our mediation analysis that elucidates a possible neurobiological pathway from PF to cognitive performance via white matter microstructure. This notion is supported by the few available experimental studies indicating that physical exercise leads to increases in memory performance and brain structural integrity^16^. This concept might be of relevance for a wide range of domains in health and life sciences including prevention, clinical care and neurobiological research. Along with previous findings, our findings point to the potential of PF as a modifiable factor that might be applied as an intervention in prevention and clinical care.

Limitations of the present work include its cross-sectional design which prevents us from inferring on causal relationships. In line with this notion, our results, especially findings from the mediation model should be interpreted with caution since they do not allow to draw any causal conclusions. Furthermore, we must acknowledge that PF was measured using a single variable while the amount, intensity and type of physical activity as well as the extent of regular physical activity or previous physical exercise of the participants was not assessed in the present study. However, the results underline the applicability of the 2-min walking test as a proxy for PF in young, healthy adults. Strengths of our study include its comparatively large and well characterized sample size and the inclusion of a wide range of relevant covariates in all main analyses. In addition, the availability of measures from several cognitive domains allowed us to delineate domain specific associations between cognition and PF as well as between cognition and white matter microstructure.

In sum, our work demonstrates that higher PF is associated with preserved white matter microstructure and better performance in a wide range of cognitive domains. The present findings thus support previous evidence for a beneficial contribution of PF on brain structure and cognitive function. The inclusion of several distinct cognitive domains and the fact that all main analyses accounted for a wide range of relevant covariates extends the present understanding of the association between PF, brain structure and cognition and supports the concept of a particularly robust association between PF and brain physiology. Future studies should aim at investigating effects of physical exercise and fitness on brain structure and cognition in longitudinal and interventional studies to ultimately clarify a potential causal effect of PF on healthy brain ageing.

## Supplementary information

Supplementary Material
