# Supplementary material for: White matter microstructure mediates the association between physical fitness and cognition in healthy, young adults
Source: Sci Rep. 2019 Sep 9;9:12885. doi: 10.1038/s41598-019-49301-y (PMC6733843; doi:10.1038/s41598-019-49301-y)
Supplement: Supplementary file 1 — Supplementary Material [file 41598_2019_49301_MOESM1_ESM.docx]

**The fast and the curious – White matter microstructure mediates the association between physical fitness and cognition in healthy, young adults**

*Supplementary Material*

Nils Opel^1^, Stella Martin^2^, Susanne Meinert^1^, Ronny Redlich^1^, Verena Enneking^1^, Maike Richter^1^, Janik Goltermann^1^, Andreas Johnen^3^, Udo Dannlowski^1^ & Jonathan Repple^1^

1 Department of Psychiatry, University of Münster, Germany

2 Department of Economics, University of Münster, Germany

3 Department of Neurology, University of Münster, Germany

Correspondence to:

Dr. Jonathan Repple, Department of Psychiatry, University of Münster, Albert-Schweitzer-Campus 1, A9, 48149 Münster, Germany, Tel: +49 251 83 566, Fax: +49 251 835 6612

E-Mail: jonathan.repple@ukmuenster.de

**Keywords:** fractional anisotropy, cognitive performance, endurance, DTI, HCP, physical fitness

Short title: Association of physical fitness with FA and cognition

**Supplementary Methods 1: Neurocognitive Tests in the HCP sample**

| **Test** | **Subdomain** | **Description** | **Score** | **Reference** |
| --- | --- | --- | --- | --- |
| NIH Toolbox Picture Sequence Memory | non-verbal episodic memory | The subject is presented a particularly ordered series of illustrated activities and objects increasing in length and recalls this order while being shown the same pictures disorderedly. | Total count of correct pairs of adjacent pictures | (Weintraub *et al*, 2013) |
| NIH Toolbox Dimensional Change Card Sort Test | executive function, cognitive flexibility | The subject matches bivalent cards to one of two target cards following either shape or color. The rule switches after several trials. | Factors in accuracy and reaction time | (Weintraub *et al*, 2013) |
| NIH Toolbox Flanker Inhibitory Control and Attention Test | executive function | The subject indicates the direction of the central one of three arrows. | Factors in accuracy and reaction time | (Weintraub *et al*, 2013) |
| Penn Progressive Matrices, total correct responses | fluid intelligence | The subject is shown matrices of 2x2, 3x3 or 1x5 squares containing one absent square and selects the most suitable out of five respond options to fill the gap. | Number of correct answers | (Bilker *et al*, 2012) |
| NIH Toolbox Oral Reading Recognition Test | reading decoding skills | The subject reads and articulates letters and words as precise as possible, while the examiner compares the response with a list of accepted pronunciations. Computer adaptive testing performs item selection. | Higher precision results in a higher score. | (Weintraub *et al*, 2013) |
| NIH Toolbox Picture Vocabulary Test | vocabulary knowledge | The subject listens to a word and picks the corresponding picture out of four response choices. Computer adaptive testing performs item selection. | A higher score displays larger vocabulary knowledge. | (Weintraub *et al*, 2013) |
| NIH Toolbox Pattern Comparison Processing Speed Test | processing speed | The subject must indicate whether two uncomplicated images, presented next to each other, are equal. | Number of correct answers given within 90 seconds | (Weintraub *et al*, 2013) |
| Delay Discounting | self-regulation/ Impulsivity | The subject chooses between a fixed delayed reward and a variable, smaller immediate amount. The first trial starts with half the delayed reward. Depending on the subject’s choice the amount is adjusted after each trial to approximate a point of equivalence after 5 trials for every combination of reward and delay. | Area-under-the-curve measure summarizing all trials for 40000$ delayed reward | (Estle *et al*, 2006; Green *et al*, 2007; Myerson *et al*, 2001) |
| Variable Short Penn Line Orientation | spatial orientation processing | The subject parallelizes two angled lines with a certain distance by rotating one of the lines. | Number of correct items | (Gur *et al*, 2001, 2010) |
| Short Penn Continuous Performance Test | sustained attention | The subject is shown vertical and horizontal lines transiently and responds, if the lines shape a number or a letter. | Specificity of right decisions | (Gur *et al*, 2001, 2010) |
| Penn Word Memory Test | verbal episodic memory | The subject tries to memorize 20 written words and to consecutively identify the previously seen words out of 40 words (the known 20 and 20 distractors). | Number of correct answers | (Gur *et al*, 2001, 2010) |
| NIH Toolbox List Sorting Working Memory Test | working memory | The subject sorts different visually or auditorily presented items by size. | Number of correct items | (Weintraub *et al*, 2013) |

**References**

Bilker WB, Hansen JA, Brensinger CM, Richard J, Gur RE, Gur RC (2012). Development of Abbreviated Nine-Item Forms of the Raven’s Standard Progressive Matrices Test. *Assessment* doi:10.1177/1073191112446655.

Estle SJ, Green L, Myerson J, Holt DD (2006). Differential effects of amount on temporal and probability discounting of gains and losses. *Mem Cogn* doi:10.3758/BF03193437.

Green L, Myerson J, Shah AK, Estle SJ, Holt DD (2007). Do Adjusting-Amount and Adjusting-Delay Procedures Produce Equivalent Estimates of Subjective Value in Pigeons? *J Exp Anal Behav* doi:10.1901/jeab.2007.37-06.

Gur RC, Ragland JD, Moberg PJ, Turner TH, Bilker WB, Kohler C, *et al* (2001). Computerized neurocognitive scanning: I. Methodology and validation in healthy people. *Neuropsychopharmacology* doi:10.1016/S0893-133X(01)00278-0.

Gur RC, Richard J, Hughett P, Calkins ME, Macy L, Bilker WB, *et al* (2010). A cognitive neuroscience-based computerized battery for efficient measurement of individual differences: Standardization and initial construct validation. *J Neurosci Methods* doi:10.1016/j.jneumeth.2009.11.017.

Myerson J, Green L, Warusawitharana M (2001). Area under the curve as a measure of discounting. *J Exp Anal Behav* doi:10.1901/jeab.2001.76-235.

Weintraub S, Dikmen SS, Heaton RK, Tulsky DS, Zelazo PD, Bauer PJ, *et al* (2013). Cognition assessment using the NIH Toolbox. *Neurology* **80**: S49–S53.

**Supplementary Figure 1: Scatterplot of the association of total endurance with total cognition**


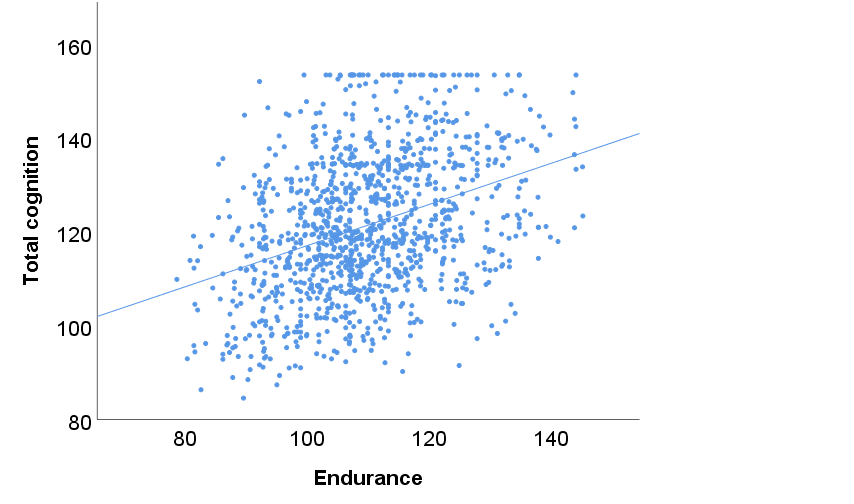


Endurance was measured with the 2-min walking test implemented in the NIH motor toolbox and cognition was assessed with the global cognition score as a composite score of all cognition subdomains. Endurance was positively associated with the global cognition score (degrees of freedom (df)= 800, standardized coefficient ß= .338, p< .001

**Supplementary Results 1: Hierarchical Regression Endurance - Cognition**

| **Descriptive Statistics** | | | |
| --- | --- | --- | --- |
|  | Mean | Std. Deviation | N |
| Total cognition | 122,1570 | 14,45479 | 801 |
| Endurance | 110,8826 | 11,83702 | 801 |
| Age | 28,75 | 3,677 | 801 |
| Sex | 1,49 | ,500 | 801 |
| Education Years | 14,95 | 1,759 | 801 |
| BMI | 26,5044 | 5,14686 | 801 |
| HbA1C | 5,2282 | ,33782 | 801 |
| BPSystolic | 123,79 | 14,141 | 801 |

| **Model Summary** | | | | | | | | | |
| --- | --- | --- | --- | --- | --- | --- | --- | --- | --- |
| Model | R | R Square | Adjusted R Square | Std. Error of the Estimate | Change Statistics | | | | |
|  |  |  |  |  | R Square Change | F Change | df1 | df2 | Sig. F Change |
| 1 | ,338^a^ | ,115 | ,113 | 13,61035 | ,115 | 103,350 | 1 | 799 | ,000 |
| 2 | ,340^b^ | ,115 | ,113 | 13,61204 | ,001 | ,801 | 1 | 798 | ,371 |
| 3 | ,341^c^ | ,116 | ,113 | 13,61476 | ,001 | ,681 | 1 | 797 | ,410 |
| 4 | ,474^d^ | ,225 | ,221 | 12,76043 | ,108 | 111,294 | 1 | 796 | ,000 |
| 5 | ,475^e^ | ,225 | ,220 | 12,76284 | ,001 | ,698 | 1 | 795 | ,404 |
| 6 | ,475^f^ | ,225 | ,219 | 12,77087 | ,000 | ,001 | 1 | 794 | ,975 |
| 7 | ,475^g^ | ,225 | ,219 | 12,77755 | ,000 | ,170 | 1 | 793 | ,680 |
| a. Predictors: (Constant), Endurance | | | | | | | | | |
| b. Predictors: (Constant), Endurance, Age | | | | | | | | | |
| c. Predictors: (Constant), Endurance, Age, Sex | | | | | | | | | |
| d. Predictors: (Constant), Endurance, Age, Sex, Education Years | | | | | | | | | |
| e. Predictors: (Constant), Endurance, Age, Sex, Education Years, BMI | | | | | | | | | |
| f. Predictors: (Constant), Endurance, Age, Sex, Education Years, BMI, HbA1C | | | | | | | | | |
| g. Predictors: (Constant), Endurance, Age, Sex, Education Years, BMI, HbA1C, BPSystolic | | | | | | | | | |

| **ANOVA^a^** | | | | | | |
| --- | --- | --- | --- | --- | --- | --- |
| Model | | Sum of Squares | df | Mean Square | F | Sig. |
| 1 | Regression | 19144,695 | 1 | 19144,695 | 103,350 | ,000^b^ |
|  | Residual | 148007,965 | 799 | 185,242 |  |  |
|  | Total | 167152,660 | 800 |  |  |  |
| 2 | Regression | 19293,177 | 2 | 9646,588 | 52,063 | ,000^c^ |
|  | Residual | 147859,483 | 798 | 185,288 |  |  |
|  | Total | 167152,660 | 800 |  |  |  |
| 3 | Regression | 19419,348 | 3 | 6473,116 | 34,922 | ,000^d^ |
|  | Residual | 147733,311 | 797 | 185,362 |  |  |
|  | Total | 167152,660 | 800 |  |  |  |
| 4 | Regression | 37541,196 | 4 | 9385,299 | 57,639 | ,000^e^ |
|  | Residual | 129611,464 | 796 | 162,828 |  |  |
|  | Total | 167152,660 | 800 |  |  |  |
| 5 | Regression | 37654,966 | 5 | 7530,993 | 46,234 | ,000^f^ |
|  | Residual | 129497,694 | 795 | 162,890 |  |  |
|  | Total | 167152,660 | 800 |  |  |  |
| 6 | Regression | 37655,129 | 6 | 6275,855 | 38,480 | ,000^g^ |
|  | Residual | 129497,531 | 794 | 163,095 |  |  |
|  | Total | 167152,660 | 800 |  |  |  |
| 7 | Regression | 37682,923 | 7 | 5383,275 | 32,972 | ,000^h^ |
|  | Residual | 129469,736 | 793 | 163,266 |  |  |
|  | Total | 167152,660 | 800 |  |  |  |
| a. Dependent Variable: Total cognition | | | | | | |
| b. Predictors: (Constant), Endurance | | | | | | |
| c. Predictors: (Constant), Endurance, Age | | | | | | |
| d. Predictors: (Constant), Endurance, Age, Sex | | | | | | |
| e. Predictors: (Constant), Endurance, Age, Sex, Education Years | | | | | | |
| f. Predictors: (Constant), Endurance, Age, Sex, Education Years, BMI | | | | | | |
| g. Predictors: (Constant), Endurance, Age, Sex, Education Years, BMI, HbA1C | | | | | | |
| h. Predictors: (Constant), Endurance, Age, Sex, Education Years, BMI, HbA1C, BPSystolic | | | | | | |

| **Coefficients^a^** | | | | | | |
| --- | --- | --- | --- | --- | --- | --- |
| Model | | Unstandardized Coefficients | | Standardized Coefficients | t | Sig. |
|  |  | B | Std. Error | Beta |  |  |
| 1 | (Constant) | 76,332 | 4,533 |  | 16,839 | ,000 |
|  | Endurance | ,413 | ,041 | ,338 | 10,166 | ,000 |
| 2 | (Constant) | 80,145 | 6,221 |  | 12,884 | ,000 |
|  | Endurance | ,409 | ,041 | ,335 | 10,015 | ,000 |
|  | Age | -,118 | ,132 | -,030 | -,895 | ,371 |
| 3 | (Constant) | 78,496 | 6,535 |  | 12,011 | ,000 |
|  | Endurance | ,419 | ,042 | ,343 | 9,873 | ,000 |
|  | Age | -,140 | ,134 | -,036 | -1,042 | ,298 |
|  | Sex | ,844 | 1,023 | ,029 | ,825 | ,410 |
| 4 | (Constant) | 55,668 | 6,496 |  | 8,569 | ,000 |
|  | Endurance | ,289 | ,042 | ,237 | 6,953 | ,000 |
|  | Age | -,254 | ,126 | -,065 | -2,010 | ,045 |
|  | Sex | -,565 | ,968 | -,020 | -,583 | ,560 |
|  | Education Years | 2,847 | ,270 | ,346 | 10,550 | ,000 |
| 5 | (Constant) | 59,332 | 7,838 |  | 7,570 | ,000 |
|  | Endurance | ,278 | ,044 | ,228 | 6,387 | ,000 |
|  | Age | -,244 | ,127 | -,062 | -1,925 | ,055 |
|  | Sex | -,735 | ,990 | -,025 | -,742 | ,458 |
|  | Education Years | 2,821 | ,272 | ,343 | 10,384 | ,000 |
|  | BMI | -,079 | ,095 | -,028 | -,836 | ,404 |
| 6 | (Constant) | 59,568 | 10,820 |  | 5,506 | ,000 |
|  | Endurance | ,278 | ,044 | ,228 | 6,354 | ,000 |
|  | Age | -,244 | ,128 | -,062 | -1,914 | ,056 |
|  | Sex | -,736 | ,991 | -,025 | -,743 | ,458 |
|  | Education Years | 2,820 | ,273 | ,343 | 10,340 | ,000 |
|  | BMI | -,079 | ,095 | -,028 | -,829 | ,407 |
|  | HbA1C | -,043 | 1,369 | -,001 | -,032 | ,975 |
| 7 | (Constant) | 61,102 | 11,446 |  | 5,338 | ,000 |
|  | Endurance | ,280 | ,044 | ,229 | 6,364 | ,000 |
|  | Age | -,247 | ,128 | -,063 | -1,935 | ,053 |
|  | Sex | -,843 | 1,025 | -,029 | -,822 | ,411 |
|  | Education Years | 2,811 | ,274 | ,342 | 10,267 | ,000 |
|  | BMI | -,065 | ,101 | -,023 | -,649 | ,516 |
|  | HbA1C | -,004 | 1,373 | ,000 | -,003 | ,998 |
|  | BPSystolic | -,015 | ,036 | -,015 | -,413 | ,680 |
| a. Dependent Variable: Total cognition | | | | | | |

**Supplementary Figure 2: Scatterplot of the association of total endurance with fractional anisotropy**


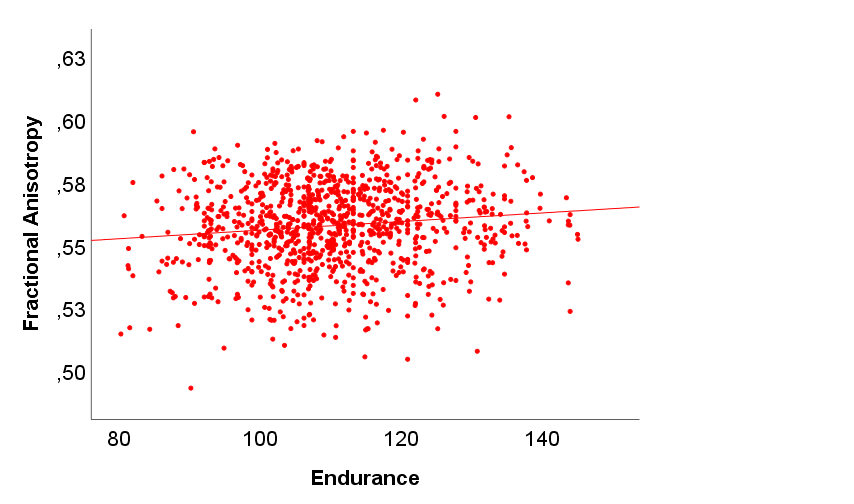


Endurance was measured with the 2-min walking test implemented in the NIH motor toolbox and fractional anisotropy is the mean value extracted from all significant voxels from the corresponding FSL analysis. We found a significant positive association (p_FWE_ < .05; cluster size k: 27313 in 4 clusters) between FA and endurance in large, widespread clusters, including the genu of the corpus callosum, the bilateral longitudinal superior fascicle, the bilateral internal and external capsule, the bilateral uncinate fascicle, the corticospinal tract and the cerebellar peduncles among others (Figure 1).

**Supplementary Results 2: Hierarchical Regression Endurance - FA**

| **Descriptive Statistics** | | | |
| --- | --- | --- | --- |
|  | Mean | Std. Deviation | N |
| FA | ,5568 | ,01702 | 724 |
| Endurance | 111,4785 | 11,76977 | 724 |
| Age | 28,66 | 3,651 | 724 |
| Sex | 1,49 | ,500 | 724 |
| Education Years | 15,01 | 1,742 | 724 |
| BMI | 25,9794 | 4,57345 | 724 |
| HbA1C | 5,2206 | ,34219 | 724 |
| BPSystolic | 123,26 | 13,702 | 724 |

| **Model Summary** | | | | | | | | | |
| --- | --- | --- | --- | --- | --- | --- | --- | --- | --- |
| Model | R | R Square | Adjusted R Square | Std. Error of the Estimate | Change Statistics | | | | |
|  |  |  |  |  | R Square Change | F Change | df1 | df2 | Sig. F Change |
| 1 | ,116^a^ | ,013 | ,012 | ,01691 | ,013 | 9,836 | 1 | 722 | ,002 |
| 2 | ,116^b^ | ,013 | ,011 | ,01692 | ,000 | ,014 | 1 | 721 | ,907 |
| 3 | ,340^c^ | ,116 | ,112 | ,01603 | ,102 | 83,306 | 1 | 720 | ,000 |
| 4 | ,341^d^ | ,116 | ,111 | ,01604 | ,000 | ,400 | 1 | 719 | ,527 |
| 5 | ,382^e^ | ,146 | ,140 | ,01578 | ,029 | 24,673 | 1 | 718 | ,000 |
| 6 | ,393^f^ | ,155 | ,148 | ,01571 | ,009 | 7,681 | 1 | 717 | ,006 |
| 7 | ,393^g^ | ,155 | ,146 | ,01572 | ,000 | ,001 | 1 | 716 | ,974 |
| a. Predictors: (Constant), Endurance | | | | | | | | | |
| b. Predictors: (Constant), Endurance, Age | | | | | | | | | |
| c. Predictors: (Constant), Endurance, Age, Sex | | | | | | | | | |
| d. Predictors: (Constant), Endurance, Age, Sex, Education Years | | | | | | | | | |
| e. Predictors: (Constant), Endurance, Age, Sex, Education Years, BMI | | | | | | | | | |
| f. Predictors: (Constant), Endurance, Age, Sex, Education Years, BMI, HbA1C | | | | | | | | | |
| g. Predictors: (Constant), Endurance, Age, Sex, Education Years, BMI, HbA1C, BPSystolic | | | | | | | | | |

| **ANOVA^a^** | | | | | | |
| --- | --- | --- | --- | --- | --- | --- |
| Model | | Sum of Squares | df | Mean Square | F | Sig. |
| 1 | Regression | ,003 | 1 | ,003 | 9,836 | ,002^b^ |
|  | Residual | ,207 | 722 | ,000 |  |  |
|  | Total | ,209 | 723 |  |  |  |
| 2 | Regression | ,003 | 2 | ,001 | 4,918 | ,008^c^ |
|  | Residual | ,207 | 721 | ,000 |  |  |
|  | Total | ,209 | 723 |  |  |  |
| 3 | Regression | ,024 | 3 | ,008 | 31,422 | ,000^d^ |
|  | Residual | ,185 | 720 | ,000 |  |  |
|  | Total | ,209 | 723 |  |  |  |
| 4 | Regression | ,024 | 4 | ,006 | 23,647 | ,000^e^ |
|  | Residual | ,185 | 719 | ,000 |  |  |
|  | Total | ,209 | 723 |  |  |  |
| 5 | Regression | ,030 | 5 | ,006 | 24,475 | ,000^f^ |
|  | Residual | ,179 | 718 | ,000 |  |  |
|  | Total | ,209 | 723 |  |  |  |
| 6 | Regression | ,032 | 6 | ,005 | 21,866 | ,000^g^ |
|  | Residual | ,177 | 717 | ,000 |  |  |
|  | Total | ,209 | 723 |  |  |  |
| 7 | Regression | ,032 | 7 | ,005 | 18,716 | ,000^h^ |
|  | Residual | ,177 | 716 | ,000 |  |  |
|  | Total | ,209 | 723 |  |  |  |
| a. Dependent Variable: FA | | | | | | |
| b. Predictors: (Constant), Endurance | | | | | | |
| c. Predictors: (Constant), Endurance, Age | | | | | | |
| d. Predictors: (Constant), Endurance, Age, Sex | | | | | | |
| e. Predictors: (Constant), Endurance, Age, Sex, Education Years | | | | | | |
| f. Predictors: (Constant), Endurance, Age, Sex, Education Years, BMI | | | | | | |
| g. Predictors: (Constant), Endurance, Age, Sex, Education Years, BMI, HbA1C | | | | | | |
| h. Predictors: (Constant), Endurance, Age, Sex, Education Years, BMI, HbA1C, BPSystolic | | | | | | |

| **Coefficients^a^** | | | | | | |
| --- | --- | --- | --- | --- | --- | --- |
| Model | | Unstandardized Coefficients | | Standardized Coefficients | t | Sig. |
|  |  | B | Std. Error | Beta |  |  |
| 1 | (Constant) | ,538 | ,006 |  | 89,823 | ,000 |
|  | Endurance | ,000 | ,000 | ,116 | 3,136 | ,002 |
| 2 | (Constant) | ,537 | ,008 |  | 66,206 | ,000 |
|  | Endurance | ,000 | ,000 | ,116 | 3,133 | ,002 |
|  | Age | 2,016E-5 | ,000 | ,004 | ,116 | ,907 |
| 3 | (Constant) | ,514 | ,008 |  | 63,393 | ,000 |
|  | Endurance | ,000 | ,000 | ,210 | 5,742 | ,000 |
|  | Age | ,000 | ,000 | -,064 | -1,774 | ,077 |
|  | Sex | ,012 | ,001 | ,342 | 9,127 | ,000 |
| 4 | (Constant) | ,512 | ,009 |  | 59,189 | ,000 |
|  | Endurance | ,000 | ,000 | ,204 | 5,354 | ,000 |
|  | Age | ,000 | ,000 | -,066 | -1,823 | ,069 |
|  | Sex | ,012 | ,001 | ,339 | 8,949 | ,000 |
|  | Education Years | ,000 | ,000 | ,023 | ,632 | ,527 |
| 5 | (Constant) | ,542 | ,010 |  | 52,225 | ,000 |
|  | Endurance | ,000 | ,000 | ,154 | 3,955 | ,000 |
|  | Age | ,000 | ,000 | -,050 | -1,406 | ,160 |
|  | Sex | ,010 | ,001 | ,300 | 7,870 | ,000 |
|  | Education Years | -1,709E-5 | ,000 | -,002 | -,048 | ,962 |
|  | BMI | -,001 | ,000 | -,183 | -4,967 | ,000 |
| 6 | (Constant) | ,568 | ,014 |  | 40,453 | ,000 |
|  | Endurance | ,000 | ,000 | ,145 | 3,736 | ,000 |
|  | Age | ,000 | ,000 | -,043 | -1,203 | ,229 |
|  | Sex | ,010 | ,001 | ,298 | 7,860 | ,000 |
|  | Education Years | ,000 | ,000 | -,011 | -,300 | ,764 |
|  | BMI | -,001 | ,000 | -,177 | -4,795 | ,000 |
|  | HbA1C | -,005 | ,002 | -,097 | -2,772 | ,006 |
| 7 | (Constant) | ,568 | ,015 |  | 38,483 | ,000 |
|  | Endurance | ,000 | ,000 | ,145 | 3,721 | ,000 |
|  | Age | ,000 | ,000 | -,043 | -1,202 | ,230 |
|  | Sex | ,010 | ,001 | ,297 | 7,603 | ,000 |
|  | Education Years | ,000 | ,000 | -,011 | -,301 | ,763 |
|  | BMI | -,001 | ,000 | -,176 | -4,506 | ,000 |
|  | HbA1C | -,005 | ,002 | -,097 | -2,760 | ,006 |
|  | BPSystolic | -1,586E-6 | ,000 | -,001 | -,033 | ,974 |
| a. Dependent Variable: FA | | | | | | |

**Supplementary Results 3: Hierarchical Regression Fractional anisotropy – Total Cognition**

| **Descriptive Statistics** | | | |
| --- | --- | --- | --- |
|  | Mean | Std. Deviation | N |
| Total cognition | 122,8919 | 14,23538 | 724 |
| FA | ,5568 | ,01702 | 724 |
| Age | 28,66 | 3,651 | 724 |
| Sex | 1,49 | ,500 | 724 |
| Education Years | 15,01 | 1,742 | 724 |
| BMI | 25,9794 | 4,57345 | 724 |
| HbA1C | 5,2206 | ,34219 | 724 |
| BPSystolic | 123,26 | 13,702 | 724 |

| **Model Summary** | | | | |
| --- | --- | --- | --- | --- |
| Model | R | R Square | Adjusted R Square | Std. Error of the Estimate |
| 1 | ,132^a^ | ,017 | ,016 | 14,12095 |
| 2 | ,145^b^ | ,021 | ,018 | 14,10543 |
| 3 | ,179^c^ | ,032 | ,028 | 14,03487 |
| 4 | ,424^d^ | ,180 | ,175 | 12,92926 |
| 5 | ,428^e^ | ,183 | ,178 | 12,90926 |
| 6 | ,428^f^ | ,183 | ,177 | 12,91804 |
| 7 | ,428^g^ | ,183 | ,176 | 12,92589 |
| a. Predictors: (Constant), FA | | | | |
| b. Predictors: (Constant), FA, Age | | | | |
| c. Predictors: (Constant), FA, Age, Sex | | | | |
| d. Predictors: (Constant), FA, Age, Sex, Education Years | | | | |
| e. Predictors: (Constant), FA, Age, Sex, Education Years, BMI | | | | |
| f. Predictors: (Constant), FA, Age, Sex, Education Years, BMI, HbA1C | | | | |
| g. Predictors: (Constant), FA, Age, Sex, Education Years, BMI, HbA1C, BPSystolic | | | | |

| **ANOVA^a^** | | | | | | |
| --- | --- | --- | --- | --- | --- | --- |
| Model | | Sum of Squares | df | Mean Square | F | Sig. |
| 1 | Regression | 2545,230 | 1 | 2545,230 | 12,764 | ,000^b^ |
|  | Residual | 143967,779 | 722 | 199,401 |  |  |
|  | Total | 146513,008 | 723 |  |  |  |
| 2 | Regression | 3060,612 | 2 | 1530,306 | 7,691 | ,000^c^ |
|  | Residual | 143452,396 | 721 | 198,963 |  |  |
|  | Total | 146513,008 | 723 |  |  |  |
| 3 | Regression | 4689,107 | 3 | 1563,036 | 7,935 | ,000^d^ |
|  | Residual | 141823,902 | 720 | 196,978 |  |  |
|  | Total | 146513,008 | 723 |  |  |  |
| 4 | Regression | 26320,789 | 4 | 6580,197 | 39,363 | ,000^e^ |
|  | Residual | 120192,220 | 719 | 167,166 |  |  |
|  | Total | 146513,008 | 723 |  |  |  |
| 5 | Regression | 26859,041 | 5 | 5371,808 | 32,234 | ,000^f^ |
|  | Residual | 119653,968 | 718 | 166,649 |  |  |
|  | Total | 146513,008 | 723 |  |  |  |
| 6 | Regression | 26863,059 | 6 | 4477,176 | 26,829 | ,000^g^ |
|  | Residual | 119649,950 | 717 | 166,876 |  |  |
|  | Total | 146513,008 | 723 |  |  |  |
| 7 | Regression | 26884,636 | 7 | 3840,662 | 22,987 | ,000^h^ |
|  | Residual | 119628,372 | 716 | 167,079 |  |  |
|  | Total | 146513,008 | 723 |  |  |  |
| a. Dependent Variable: Total cognition | | | | | | |
| b. Predictors: (Constant), FA | | | | | | |
| c. Predictors: (Constant), FA, Age | | | | | | |
| d. Predictors: (Constant), FA, Age, Sex | | | | | | |
| e. Predictors: (Constant), FA, Age, Sex, Education Years | | | | | | |
| f. Predictors: (Constant), FA, Age, Sex, Education Years, BMI | | | | | | |
| g. Predictors: (Constant), FA, Age, Sex, Education Years, BMI, HbA1C | | | | | | |
| h. Predictors: (Constant), FA, Age, Sex, Education Years, BMI, HbA1C, BPSystolic | | | | | | |

| **Coefficients^a^** | | | | | | |
| --- | --- | --- | --- | --- | --- | --- |
| Model | | Unstandardized Coefficients | | Standardized Coefficients | t | Sig. |
|  |  | B | Std. Error | Beta |  |  |
| 1 | (Constant) | 61,498 | 17,192 |  | 3,577 | ,000 |
|  | FA | 110,263 | 30,862 | ,132 | 3,573 | ,000 |
| 2 | (Constant) | 68,283 | 17,683 |  | 3,861 | ,000 |
|  | FA | 109,981 | 30,829 | ,131 | 3,567 | ,000 |
|  | Age | -,231 | ,144 | -,059 | -1,609 | ,108 |
| 3 | (Constant) | 56,232 | 18,087 |  | 3,109 | ,002 |
|  | FA | 135,141 | 31,898 | ,162 | 4,237 | ,000 |
|  | Age | -,133 | ,147 | -,034 | -,904 | ,366 |
|  | Sex | -3,200 | 1,113 | -,112 | -2,875 | ,004 |
| 4 | (Constant) | 27,848 | 16,848 |  | 1,653 | ,099 |
|  | FA | 108,712 | 29,477 | ,130 | 3,688 | ,000 |
|  | Age | -,266 | ,136 | -,068 | -1,960 | ,050 |
|  | Sex | -3,629 | 1,026 | -,128 | -3,537 | ,000 |
|  | Education Years | 3,170 | ,279 | ,388 | 11,376 | ,000 |
| 5 | (Constant) | 40,910 | 18,325 |  | 2,233 | ,026 |
|  | FA | 96,572 | 30,197 | ,115 | 3,198 | ,001 |
|  | Age | -,246 | ,136 | -,063 | -1,808 | ,071 |
|  | Sex | -3,752 | 1,027 | -,132 | -3,655 | ,000 |
|  | Education Years | 3,070 | ,284 | ,376 | 10,820 | ,000 |
|  | BMI | -,200 | ,111 | -,064 | -1,797 | ,073 |
| 6 | (Constant) | 39,458 | 20,587 |  | 1,917 | ,056 |
|  | FA | 97,107 | 30,413 | ,116 | 3,193 | ,001 |
|  | Age | -,248 | ,137 | -,064 | -1,813 | ,070 |
|  | Sex | -3,758 | 1,028 | -,132 | -3,655 | ,000 |
|  | Education Years | 3,075 | ,286 | ,376 | 10,766 | ,000 |
|  | BMI | -,201 | ,112 | -,065 | -1,802 | ,072 |
|  | HbA1C | ,223 | 1,435 | ,005 | ,155 | ,877 |
| 7 | (Constant) | 38,000 | 20,995 |  | 1,810 | ,071 |
|  | FA | 96,985 | 30,434 | ,116 | 3,187 | ,002 |
|  | Age | -,245 | ,137 | -,063 | -1,788 | ,074 |
|  | Sex | -3,649 | 1,073 | -,128 | -3,400 | ,001 |
|  | Education Years | 3,078 | ,286 | ,377 | 10,766 | ,000 |
|  | BMI | -,215 | ,118 | -,069 | -1,822 | ,069 |
|  | HbA1C | ,188 | 1,440 | ,005 | ,130 | ,896 |
|  | BPSystolic | ,014 | ,040 | ,014 | ,359 | ,719 |
| a. Dependent Variable: Total cognition | | | | | | |
